# SARS-CoV-2 infection is associated with a pro-thrombotic platelet phenotype

**DOI:** 10.1038/s41419-020-03333-9

**Published:** 2021-01-05

**Authors:** Dario Bongiovanni, Melissa Klug, Olga Lazareva, Simon Weidlich, Marina Biasi, Simona Ursu, Sarah Warth, Christian Buske, Marina Lukas, Christoph D. Spinner, Moritz von Scheidt, Gianluigi Condorelli, Jan Baumbach, Karl-Ludwig Laugwitz, Markus List, Isabell Bernlochner

**Affiliations:** 1 Department of Internal Medicine I, School of Medicine, University hospital rechts der Isar, Technical University of Munich, Munich, Germany; 2 German Center for Cardiovascular Research (DZHK), Partner Site Munich Heart Alliance, Munich, Germany; 3 Department of Cardiovascular Medicine, Humanitas Clinical and Research Center IRCCS and Humanitas University, Rozzano, Milan, Italy; 4 Experimental Bioinformatics, TUM School of Life Sciences Weihenstephan, Technical University of Munich, Munich, Germany; 5 Department of Internal Medicine II, School of Medicine, University hospital rechts der Isar, Technical University of Munich, Munich, Germany; 6 Core Facility Cytometry, Ulm University Medical Faculty, Ulm, Germany; 7 CCC Ulm, Institute of Experimental Cancer Research, University Hospital Ulm, Ulm, Germany; 8 Deutsches Herzzentrum München, Cardiology, Technical University of Munich, Munich, Germany

**Keywords:** Mechanisms of disease, Viral infection

## Abstract

Novel coronavirus disease 2019 (COVID-19) is associated with a hypercoagulable state, characterized by abnormal coagulation parameters and by increased incidence of cardiovascular complications. With this study, we aimed to investigate the activation state and the expression of transmembrane proteins in platelets of hospitalized COVID-19 patients. We investigated transmembrane proteins expression with a customized mass cytometry panel of 21 antibodies. Platelets of 8 hospitalized COVID-19 patients not requiring intensive care support and without pre-existing conditions were compared to platelets of healthy controls (11 donors) with and without in vitro stimulation with thrombin receptor-activating peptide (TRAP). Mass cytometry of non-stimulated platelets detected an increased surface expression of activation markers P-Selectin (0.67 vs. 1.87 median signal intensity for controls vs. patients, *p* = 0.0015) and LAMP-3 (CD63, 0.37 vs. 0.81, *p* = 0.0004), the GPIIb/IIIa complex (4.58 vs. 5.03, *p* < 0.0001) and other adhesion molecules involved in platelet activation and platelet–leukocyte interactions. Upon TRAP stimulation, mass cytometry detected a higher expression of P-selectin in COVID-19 samples compared to controls (*p* < 0.0001). However, we observed a significantly reduced capacity of COVID-19 platelets to increase the expression of activation markers LAMP-3 and P-Selectin upon stimulation with TRAP. We detected a hyperactivated phenotype in platelets during SARS-CoV-2 infection, consisting of highly expressed platelet activation markers, which might contribute to the hypercoagulopathy observed in COVID-19. In addition, several transmembrane proteins were more highly expressed compared to healthy controls. These findings support research projects investigating antithrombotic and antiplatelet treatment regimes in COVID-19 patients, and provide new insights on the phenotypical platelet expression during SARS-CoV-2 infection.

## Introduction

Despite severe acute respiratory syndrome coronavirus 2 (SARS-CoV-2)’s worldwide spread, little is known about the pathophysiological mechanisms leading to multiorgan damage in coronavirus disease 2019 (COVID-19). A hypercoagulable state with increased incidence of cardiovascular complications and venous thrombotic events has been reported in several studies^1–7^. Abnormal coagulation parameters are observed in hospitalized patients and are associated with poor prognosis^8–10^. Interestingly, two studies reported in COVID-19 alterations in platelet transcriptome and proteome, and an increased platelet reactivity^11,12^. A recent study described the presence microvascular thrombi in lung, heart and kidney containing neutrophil extracellular traps (NETs) in severe SARS-CoV-2 infection, as well as circulant neutrophil-platelet aggregates and immunothrombotic dysregulation, which changes with disease severity^13^. Recently, a computational system’s medicine platform identified as new drug target several proteins involved in the coagulation cascade^14,15^. However, the role of platelet activation and changes of transmembrane receptor expression in COVID-19-induced coagulopathy still needs to be further investigated.

Platelets not only play a pivotal role in vascular hemostasis but are also involved in immune response, tumor progression, and other inflammatory processes^16^. They are activated during sepsis and in septic shock, and antiplatelet therapy has been suggested as a novel strategy to prevent organ damage^17^. In fact, in the presence of severe infections or cytokine storms^18,19^, platelet hyperreactivity may be responsible for major cardiovascular adverse events^20^. Viral infections are known to be associated with coagulation disorders^21^. Interestingly, an increased incidence of acute coronary syndrome has been observed after influenza infection^22^, suggesting that viral diseases could trigger platelet activation leading to cardiovascular complications. Moreover, viral-induced coagulopathies have been already observed in SARS-CoV-1 infection including thrombocytosis, disseminated intravascular coagulation, and thromboembolism^23,24^. In this study, we investigated the expression of platelet transmembrane receptors and adhesion molecules at baseline level and after in vitro platelet stimulation in hospitalized COVID-19 patients without pre-existing conditions and in healthy donors using mass cytometry by time of flight (CyTOF). Here we present the largest existing CyTOF panel of platelet antibodies specifically developed to investigate platelet activation and adhesion (Table 1).

**Table 1:** Mass cytometry panel.

Antigen	Common name	Biological function
CD107a	LAMP-1	Cell adhesion, activation marker
CD141	Thrombomodulin	Thrombin-binding protein
CD154	CD40L, CD40 ligand	Regulation of platelet–leukocyte interactions
CD29	Integrin subunit β1	Fibronectin and collagen receptor subunit
CD3	TCR–CD3 complex	Adaptive immune response, negative control
CD31	PECAM-1	Cell adhesion
CD36	GPIV	Thrombospondin receptor, cell adhesion
CD40	TNFRSF5	Mediates immune and inflammatory responses
CD41	Integrin αIIb, GPIIb	α-Unit of fibrinogen receptor
CD42a	GPIX	Von Willebrand factor receptor unit
CD42b	GPIbα	Von Willebrand factor receptor unit
CD47	MER6	adhesion receptor for THBS1 on platelets
CD61	Integrin β3, GPIIIa	β-Unit of fibrinogen receptor
CD62P	P-Selectin	Cell adhesion, activation marker
CD63	LAMP-3	Cell adhesion, platelet activation marker
CD69	CLEC2C	Signal transmission in NKCs and platelets
CD9	Tetraspanin-29	Cell adhesion, integrin binding
F2R	Par1	Thrombin receptor
GPVI	Platelet glycoprotein 6	Collagen receptor
GPIIbIIIa	GPIIb/GPIIIa complex	GPIIb/GPIIIa complex-specific antibody
PEAR1	JEDI	Platelet endothelial aggregation receptor

## Methods

### Data and code availability

All mass cytometry data have been made available at flowrepository.org and can be accessed at repository ID FR-FCM-Z2MT. The scripts used in this analysis have been deposited at github.com and can be accessed at https://github.com/biomedbigdata/SARS-CoV-2-platelets-analysis.

### Study design and participants

SARS-CoV-2-infected patients hospitalized at the Klinikum recht der Isar, Munich, Germany, between March and May 2020 with symptomatic COVID-19 not requiring intensive care unit admission and without known pre-existing conditions were recruited in our study and compared to an asymptomatic control cohort of healthy donors. Inclusion criteria for the COVID-19 group were a symptomatic (dyspnea) SARS-CoV-2 infection confirmed by a positive reverse-transcription PCR assay from any respiratory specimen or IgM antibodies in peripheral blood, age between >18 and <70 years, and written informed consent.

Exclusion criteria were known platelet dysfunctions, relevant thrombocytopenia (<100 G/l) or thrombocytosis (>500 G/l), impaired renal function (glomerular filtration rate < 60ml/min), hemoglobin < 10g/dl, leukocytes < 1 G/l, any known pre-existing condition except arterial hypertension, any medication except antihypertensive drugs, and a history of hematological neoplasia including active lymphoma, mental impairment, or pregnancy.

Blood samples were collected from patients within the first 36 h after admission. As a control group, we recruited a healthy and asymptomatic cohort of donors. All healthy donors were tested negative for SARS-CoV-2 IgG and IgM, were followed up, and did not develop any symptoms in the weeks following the recruitment. Throughout the entire study design, patients’ samples were handled together with control samples. The study complied to the Declaration of Helsinki, was approved by the local ethics committee (approval numbers 147/20 and 352/18), and all participants provided written informed consent.

### Sample collection and preparation

Peripheral venous blood was collected in citrate tubes and immediately processed to produce platelet-rich plasma (PRP) as described before^25,26^. CyTOF staining assay was performed according to the manufacturer’s protocols. Briefly, 600 µl PRP previously inhibited by a mixture of 0.6 U Apyrase/ml, 20 mM of HEPES, and 1 mM EGTA was diluted in phosphate-buffered saline (PBS) pursuant to the gold standard protocol for mass cytometry (Fluidigm, San Francisco, CA, USA) to a final concentration of 10^5^ platelets/µl. The PRP was stained with 5 µM Cell-ID^TM^ Cisplatin (Fluidigm) for 5 min and then washed with 5 ml MaxPar Cell Staining Buffer (Fluidigm). After centrifugation, cells were resuspended in 50 µl Cell Staining Buffer. Two samples were prepared from each donor: one baseline sample (non-stimulated platelets) and one sample stimulated with 10 µM thrombin receptor-activating peptide (TRAP). TRAP addition was followed by a 2 min incubation at room temperature. In the same cell suspensions platelets were stained with 50 µl of the custom-made CyTOF-antibody panel in Cell Staining Buffer for 30 min (containing anti-CD3-170Er, anti-CD9-171Yb, anti-CD29-156Yb, anti-CD31-145Nd, anti-CD36-152Nd, anti-CD40-142Nd, anti-CD41-89Y, anti-CD42a-141Pr, anti-CD42b-144Nd, anti-CD47-209Bi, anti-CD61-146Nd, anti-CD62P-161Dy, anti-CD63-150Nd, anti-CD69-162Dy, anti-CD107a-151Eu, anti-CD141-166Er, anti-CD154-168Er, anti-GPVI-175Lu, anti-GPIIb/GPIIIa complex-155Gd, anti-Par1-147Sm, and anti-PEAR-147Sm; see Supplemental Material for antibody information). After washing twice with 2 ml Cell Staining Buffer at 300 g for 5 min, cells were fixed overnight at 4 °C in 1 ml of 1.6% Formaldehyde. After fixation, cells were pelleted at 800 × *g* for 10 min, the supernatant was aspirated and removed. Then, cells were gently vortexed and resuspended in the residual volume (~50 µl) and incubated with 125 nM Iridium in 1 ml MaxPar Fix and Perm Buffer (Fluidigm) for 1 h following the manufacturer’s protocol (Fluidigm). Afterwards, they were centrifuged at 800 × *g* for 5 min, then washed with 2 ml Cell Staining Buffer at 800 g for 5 min, then frozen in 10% dimethyl sulfoxide (DMSO) in fetal bovine serum until acquisition^27^. After thawing the samples, they were washed twice with Cell Staining Buffer and once with water at 800 × *g* for 5 min to eliminate DMSO remnants. Cells were then handled according to the manufacturer’s protocol. Prior to measurement, cells were diluted to a final concentration of 10^3^ platelets/µl before addition of EQ calibration beads. Cells were measured using a Helios mass cytometer (Fluidigm). Throughout the study, patients’ samples were measured with at least one control sample to reduce batch effect. In total, 476,756 ± 151,746 events were acquired at a rate of 300–500 events per second. Experiments were carried out by the same scientist and antibodies were from the same lot. See Supplemental Material for a complete reagent list.

### Mass cytometry

CyTOF allows multidimensional relative protein quantification for single-cell datasets and we adapted it for platelets using a customized mass cytometry panel of 21 antibodies (Table 1). For custom-made antibody conjugations, 100 mg of carrier-free antibody was coupled to metal-labeled X8 polymer according to the manufacturer’s instructions (Fluidigm). Briefly, using the MaxPAR antibody conjugation kit (Fluidigm) following the manufacturer’s recommended protocol, six antibodies were conjugated to isotopically enriched lanthanide metals. After labeling, the antibodies were stored in an antibody stabilization buffer (Boca Scientific, Westwood, MA, USA) at 4 °C. The other antibodies were pre-conjugated, CyTOF-ready, and commercially available (Fluidigm Sciences). Please see the Supplemental Materials for the reagent list. All custom-conjugated antibodies were validated with calibration beads. In detail, 0.5 µl of the conjugated antibody was added to one drop of beads and incubated for 15 min. After two washing steps with 1.5 ml PBS at 300 × *g* for 10 min, the mixture was washed twice with de-ionized water at 300 × *g* for 10 min, and resuspended in 200 µl water until acquisition.

### CyTOF processing

After acquisition, samples were cleaned up according to the latest standard of data pre-gating (Fluidigm) using the Cytobank™ software (www.cytobank.org, Beckman-Coulter, Brea, CA, USA)^28^. To avoid leukocyte contamination, we gated the acquired events for platelet-specific markers: only CD41 (GPIIb)- and CD61-(GPIIIa) positive events were selected for further analysis and defined as platelets (Supplemental Fig. I). CD3 marker was included in the panel as an additional negative control (Supplemental Fig. II).

### Computational analysis

**Fig. 1:**
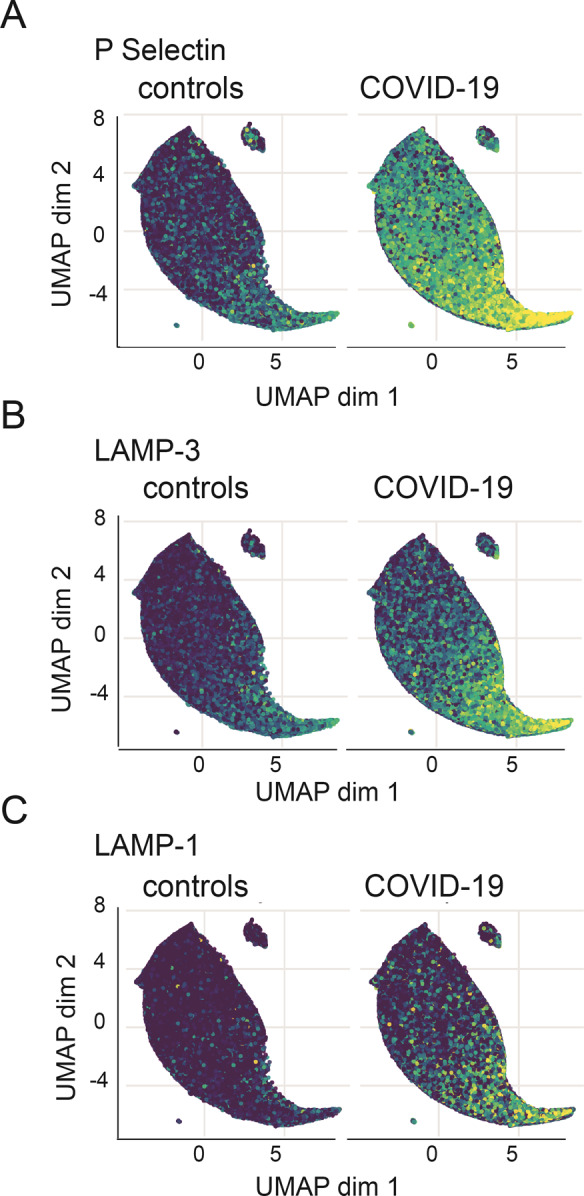
Activation marker expression in non-stimulated platelets. Uniform manifold approximation and projection (UMAP) after equal random sampling from each sample and scaled, arcsinh-transformed expression [0–1] for each activation marker colored according to the expression level: **A** P-Selectin, **B** LAMP-3, and **C** LAMP-1, *N* = 8 COVID-19 patients, 11 healthy donors.

All models were built and assessed using the statsmodels v0.11.1 python package^29^. CyTOF data were processed using Cytobank and analyzed using R 4.0 (R Development Core Team, 2005) and Python 3.6 (Python 3 Reference Manual, 2009). For visualization of activation markers in reduced dimensions, we performed uniform manifold approximation and projection based on 16 markers (excluding the activation markers) using the CATALYST v1.12.1 R package. To account for differences in coverage between samples, we randomly sampled the minimum number of events acquired (41,525 events per sample, Fig. 1). Following standard practice for differential marker expression in CyTOF^30^, we built mixed-effect linear models for the TRAP-stimulated and non-stimulated sample groups, respectively. We considered the sample-wise median signal intensity as dependent variable, disease status as fixed and patient IDs as random effect, i.e., each patient has a different intercept. Furthermore, we built a linear model for all samples (TRAP-stimulated and non-stimulated) with an interaction term to assess whether activation is significantly affected by disease status. *P*-values of model coefficients were corrected for multiple hypothesis testing using the Benjamini–Hochberg method (false discovery rate < 0.05)^31^.

### Differential analysis of overall marker expression

Statistical significance is evaluated based on regression analysis. To estimate whether the sample-wise median expression of a marker is significantly different between COVID-19 patients and healthy controls, the following linear mixed-effect model was used:

1 $${{Y}}_{{{ij}}} = {\beta}_{0{{j}}} + {\beta}_{{{cj}}}\,{{x}}_{{{ci}}} + {\gamma}_{{i}} + \epsilon _{{{ij}}}$$

where *Y*_*ij*_ is the median expression of the *j*-th marker for *i*-th patient, *x*_*ci*_ is a binary variable indicating if a patient *i* belongs to case or control group, and $\gamma _i \sim N\left( {0,\sigma _i^2} \right)$ is a random intercept for each patient. The latter allows us to disentangle within-sample and within-group variance.

Slope coefficients $\beta _{cj}$ were tested for significance of the linear relationship between the independent variable *x*_*c*_ and the dependent variable *Y*:

2 $${{H}}_0:{\beta}_{{{cj}}} = 0$$

$${{H}}_1:{\beta}_{{{cj}}}\, \ne\, 0$$

### Difference in TRAP stimulation effect for COVID-19 patients

To analyze if a higher expression of activation markers in COVID-19 quiescent platelets is coupled with a reduced capacity to react upon activation stimuli, we compared slope coefficients for the covariate that corresponds to activation for cases and controls separately. We used the following model for healthy controls:

3 $${{Y}}_{{{hj}}} = {\beta}_{0{{j}}} + {\beta}_{{{aj}}}{{x}}_{{{ah}}} + {\gamma}_{{h}} + \epsilon _{{{hj}}}$$

where *h* belongs to a set of indices for all healthy controls and *x*_*ah*_ is a binary variable that indicates if a sample *h* was TRAP-stimulated or not. We computed a set of slopes $\beta _{aj}$ that show an average linear increase in expression after activation for every marker *j*.

A similar model was used for COVID-19 patients:

4 $${{Y}}_{{{pj}}} = {\beta}_{0{{j}}} + {\beta}_{{{aj}}}{{x}}_{{{ap}}} + {\gamma}_{{p}} + \epsilon _{{{pj}}}$$

where *p* belongs to a set of indices for all COVID-19 patients. Although we can compare $\beta _a$ coefficients from the model (3) and model (4) directly, we cannot conclude if the difference between slope coefficients is statistically significant. To evaluate if there is a statistically significant difference in the reaction to TRAP stimulation between patients and controls, we used a single model with an interaction effect term. Significance of the Interaction effect means that activation status and patient condition (disease or control) combined have a significantly larger effect on median signal intensity as compared to the sum of the individual factors alone. Formally, this results in the following model:

5 $${{Y}}_{{{ij}}} = {\beta}_0 + {\beta}_{{a}}{{x}}_{{{aij}}} + {\beta}_{{c}}{{x}}_{{{cij}}} + {\beta}_{{{intij}}}{{x}}_{intij} + {\gamma}_{{i}} + \epsilon _{{{ij}}}$$

where *x*_*int*_ is an interaction term defined as *x*_*a*_ × *x*_*c*_. The slope coefficient for the interaction ${\beta}_{int}$ was then tested for statistical significance as shown in Eq. (2).

### Clustering analysis: FlowSOM algorithm

Automated clustering analysis was done using the FlowSOM algorithm^32^. After gating (Supplemental Fig. I), data were compensated, transformed with an estimated logical transformation, and scaled. Cells were assigned to a 10 × 10 self-organizing map and then metaclustering in 12 clusters was performed. The number of clusters was selected based on relative change in area under the cumulative distribution function curve that indicated that cells stratification in more than 12 clusters cannot improve the clustering results. For each cluster-marker pair, a *p*-value was computed using a linear mixed-effect model:

6 $${{Y}}_{{{ij}}} = {\beta}_0 + {\beta}_{{{cj}}}{{x}}_{{{ci}}} + {\gamma}_{{i}} + \epsilon _{{{ij}}}$$

where *Y*_*ij*_ is the median expression of the *j*-th marker for *i*-th cluster, *x*_*ci*_ is a binary variable indicating if cell population *Y*_*ij*_ belongs to case or control group, and $\gamma _i \sim N\left( {0,\sigma _i^2} \right)$ is a random intercept for each cell subpopulation. Slope coefficients ${\beta}_{{{cj}}}$were tested for significance of the linear relationship between the independent variable *x*_*c*_ and the dependent variable *Y*.

## Results

### Study population characteristics

Eight hospitalized symptomatic COVID-19 patients without pre-existing conditions requiring oxygen support were recruited and compared to a cohort of 11 asymptomatic healthy donors, tested negative for SARS-CoV-2 (mean age COVID-19: 51.4 ± 11.7, controls: 44.7 ± 13.0, *p* = 0.27; male COVID-19 62.5%, controls: 45% *p* = 0.49). Seven patients showed typical COVID-19 pulmonary lesions in chest computed tomography. Patients were admitted through the emergency department and moved to normal wards due to dyspnea. During the hospitalization, one patient was transferred to an intermediated care unit (2 days after blood collection) for a few hours, for the purpose of monitoring due to respiratory deterioration. No patient required assisted ventilation and all were discharged in good condition (average hospitalization 9.5 ± 6.3 days). No major adverse events (bleeding and thromboembolic events) were reported. All admitted patients were not under regular medication, except one with two antihypertensive medications: amlodipine and valsartan. For a detailed description of the study population, see the Supplementary Table 1.

### Platelet surface receptor and adhesion molecule expression in non-stimulated platelets

**Fig. 2:**
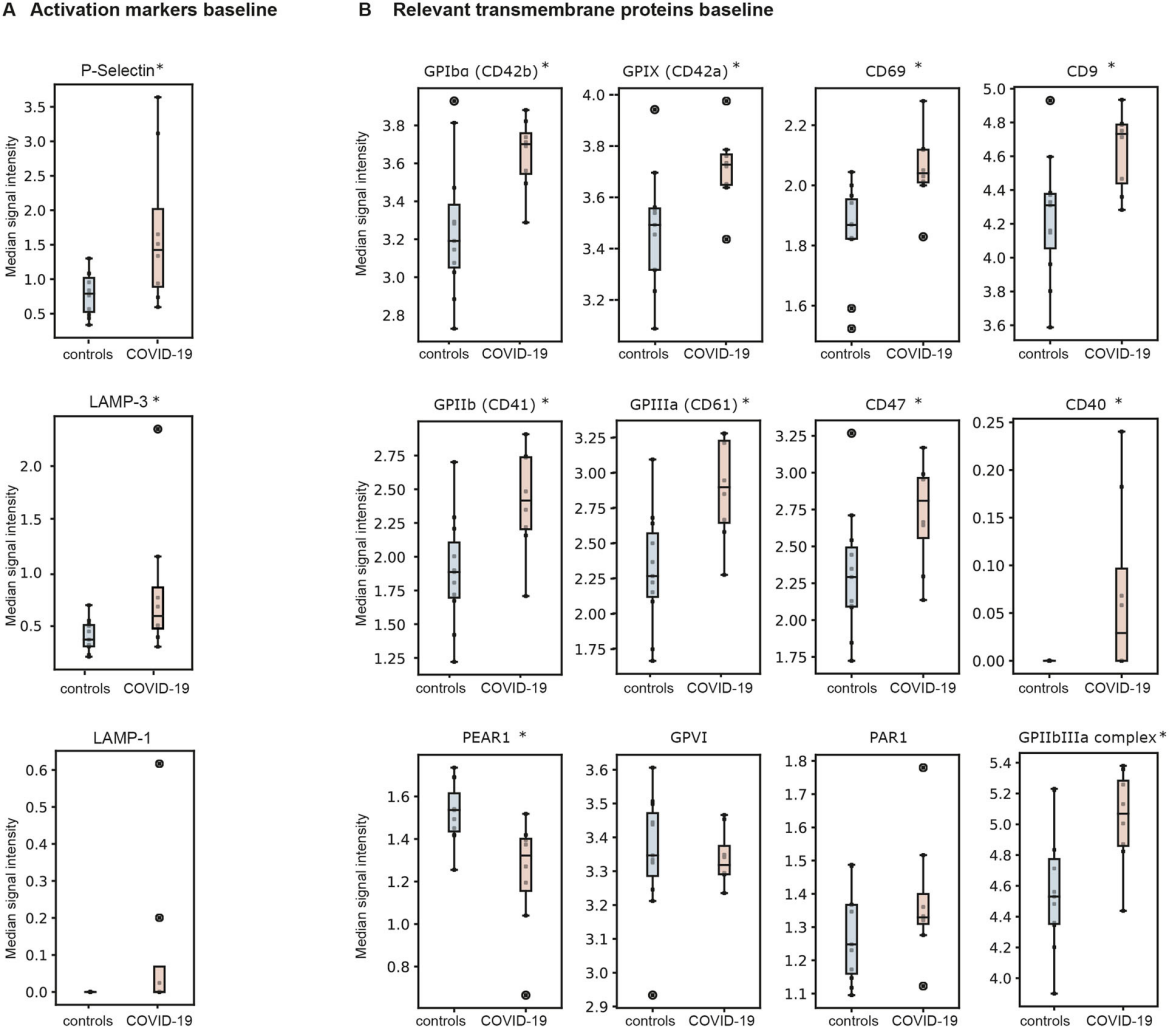
Marker expression in non-stimulated platelets. Median signal intensity of activation markers (**A**) and relevant transmembrane proteins (**B**) in non-stimulated platelets. COVID-19 patients are plotted in red, whereas controls are plotted in blue. The horizontal line within the box plot represents the median, the top and bottom the interquartile range(Q1–Q3), whisker bars indicate the largest observation that is less than or equal to the upper inner fence (UIF = Q3 + 1.5 × IQR) or the smallest observation that is greater than or equal to the lower inner fence (LIF = Q1–1.5 × IQR) and the circles indicate outliers, if present; *P < 0.01. *N* = 8 COVID-19 patients, 11 healthy donors.

Compared to healthy controls, non-stimulated platelets of COVID-19 patients showed a significantly higher spontaneous expression of specific platelet activation markers (Fig. 1), such as P-Selectin (0.67 vs. 1.87 median signal intensity for healthy donors vs. patients, *p* = 0.0015) and LAMP-3 (0.37 vs. 0.81 median signal intensity, *p* = 0.0004, Fig. 2a), as well as the the GPIIb/IIIa complex (4.58 vs. 5.03 median signal intensity, *p* < 0.0001). In addition, we detected a higher spontaneous expression of some constitutive receptors and adhesion molecules involved in platelet activation and aggregation in COVID-19 platelets, such as the transmembrane integrins GPIIb (*p* = 0.0001) and GPIIIa (*p* < 0.0001), as well as the glycoproteins GPIbα (*p* = 0.0086) and GPIX (*p* = 0.0126, Fig. 2b). The expression level of all other activation markers and adhesion molecules are shown in Supplemental Fig. II.

### Diseased platelet reactivity after TRAP stimulation

**Fig. 3:**
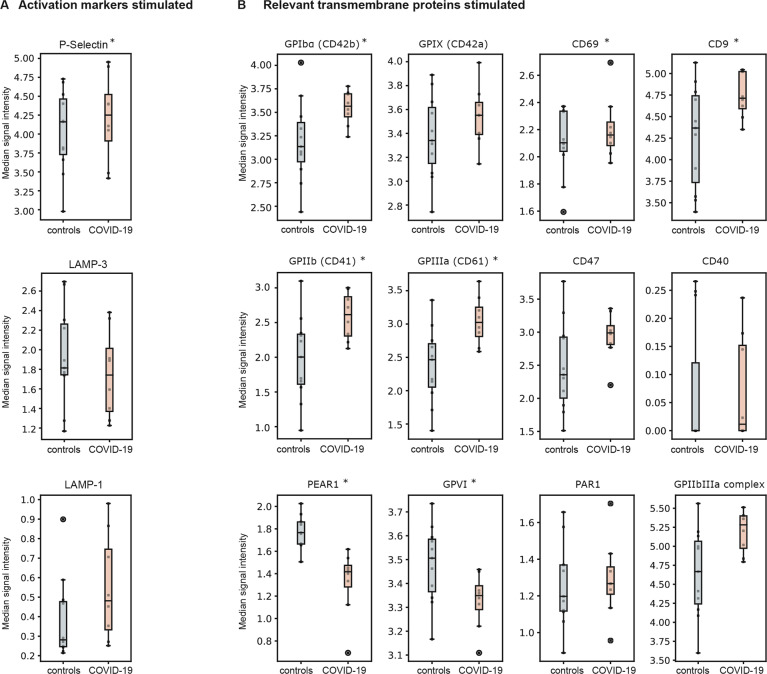
Marker expression in TRAP-stimulated platelets. Median signal intensity of activation markers (**A**) and relevant transmembrane proteins (**B**) in TRAP-stimulated platelets (10 µM TRAP). COVID-19 patients are plotted in red, whereas controls are plotted in blue. The horizontal line within the box plot represents the median, the top and bottom the interquartile range (Q1–Q3), whisker bars indicate the largest observation that is less than or equal to the upper inner fence (UIF = Q3 + 1.5 × IQR) or the smallest observation that is greater than or equal to the lower inner fence (LIF = Q1–1.5 × IQR) and the circles indicate outliers, if present; *P < 0.01. *N* = 8 COVID-19 patients, 11 healthy donors.

To further investigate platelet reactivity, we stimulated the collected platelets with 10 µM TRAP. Upon TRAP stimulation, mass cytometry also detected a significantly higher expression of the platelet activation marker P-selectin in samples of COVID-19 patients compared to healthy controls (*p* = 0.0176), but LAMP-3 did not show significant differences (*p* = 0.40, Fig. 3a). Interestingly, the GPIIb/GPIIIa complex remained upregulated in COVID-19 patients after TRAP stimulation (*p* < 0.0001). Similar to non-stimulated platelets, we also observed a higher expression level for the integrins GPIIb (*p* < 0.0001), GPIIIa (*p* = 0.0009), as well as for the glycoproteins GPIbα in TRAP-stimulated platelets compared to healthy controls (*p* < 0.0001, Fig. 3b). In Table 2, we provide a complete result list of markers tested.

**Table 2:** Median signal intensity and *p*-values of CyTOF panel.

	Non-stimulated			TRAP-stimulated		
	Controls	COVID-19	*p*-Value	Controls	COVID-19	*p*-Value
CD41	1.8856	2.3906	0.0001	1.877	2.5435	<0.0001
CD40	0	0.0308	0.0005	0	0.0612	0.915
CD42b	3.2253	3.6501	0.0086	3.1198	3.4849	<0.0001
CD31	2.2122	2.5058	0.0522	2.1849	2.5	0.2388
CD61	2.3059	2.8594	<0.0001	2.2974	2.9396	0.0009
PAR1	1.2734	1.4094	0.1697	1.2733	1.3221	0.7218
CD63	0.347	0.8061	0.0004	1.9609	2.0531	0.4026
CD107a	0	0.0322	1	0.3622	0.6348	0.2114
CD36	2.928	3.2467	0.4988	2.8899	3.2625	0.2388
GPIIb/GPIIIa complex	4.5825	5.0363	<0.0001	4.5809	5.1493	0.0176
CD29	3.8587	4.0407	0.0761	3.8499	4.0422	0.2077
CD62P	0.6714	1.8705	0.0015	4.0868	4.3855	<0.0001
CD69	1.8559	2.0478	0.0002	2.1615	2.2371	0.0326
CD141	0	0	1	0	0	0.3288
CD154	0	0	1	0.2288	0.2188	0.8033
CD3	0	0	1	0	0	1
CD9	4.2401	4.6766	0.001	4.2612	4.7279	0.0176
PEAR	1.5399	1.2474	0.0039	1.8075	1.41	<0.0001
GPVI	3.3737	3.3506	0.4884	3.4984	3.3675	0.0176
CD47	2.3005	2.7849	0.0015	2.3625	2.9007	0.0555
CD42a	3.4665	3.7047	0.0126	3.3374	3.5062	0.2077

**Fig. 4:**
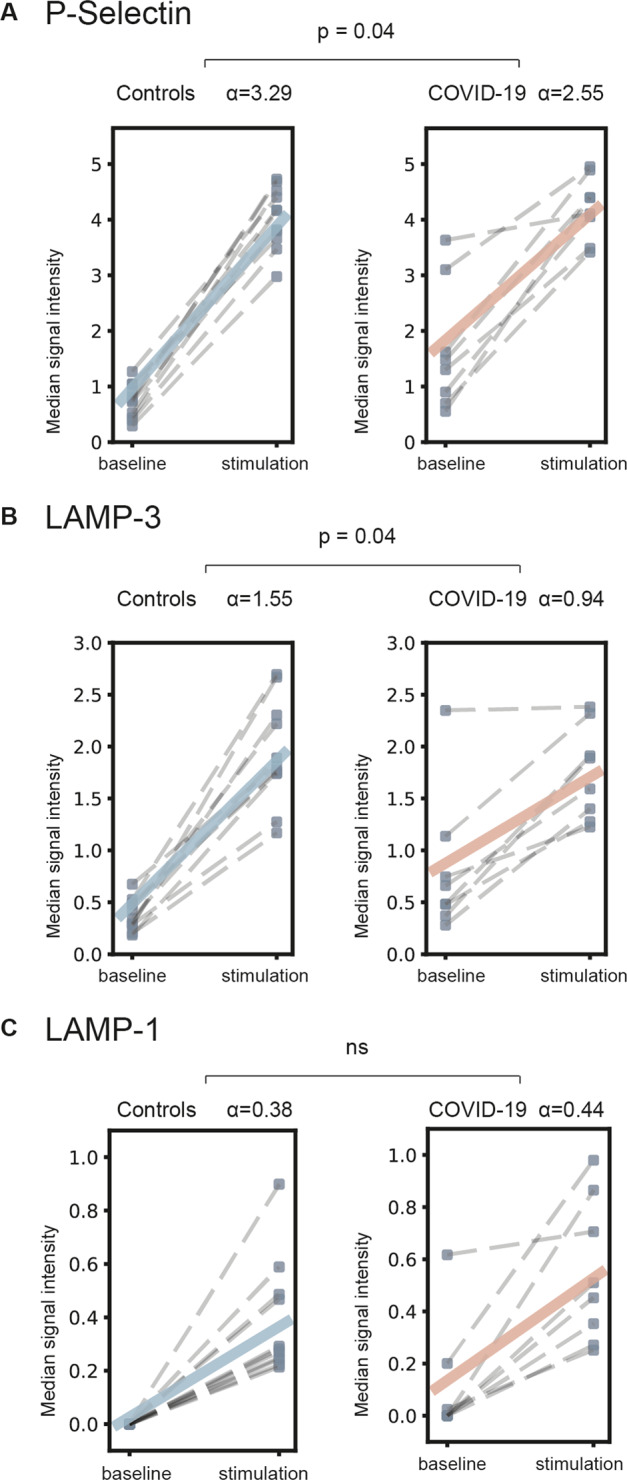
Platelet reaction capacity. Median signal intensity increase of activation marker expression after TRAP stimulation (10 µM) compared to non-stimulated platelets (baseline). Linear model analysis detected a reduced capacity of COVID-19 patients to increase expression of activation markers LAMP-3 and P-Selectin (*p* = 0.04 and *p* = 0.04) after TRAP stimulation. **A** P-Selectin, **B** LAMP-3, **C** LAMP-1. α: signal increment slope coefficient (for details see Methods); ns: nonsignificant. *N* = 8 COVID-19 patients, 11 healthy donors.

To assess the reaction capacity of platelets upon stimulation, we compared the expression of activation markers before and after stimulation with TRAP. Interestingly, we observed a significantly reduced capacity of COVID-19 platelets to increase the expression of the activation markers LAMP-3 and P-Selectin (*p* = 0.04 and *p* = 0.04, respectively) upon stimulation (Fig. 4).

## Discussion

We analyzed the expression of activation markers and transmembrane receptors in platelets of hospitalized stable COVID-19 patients without pre-existing conditions and without anticoagulants or antiplatelet drugs (except prophylactic low-molecular-weight heparin during hospitalization). As a major result, we detected significant higher levels of the platelet activation markers P-Selectin and LAMP-3 compared to controls, as well as significantly higher levels of the transmembrane proteins GPIIb/GPIIIa complex, GPIbα, GPIX, CD9, and CD40. After TRAP stimulation, platelets of COVID-19 patients showed significantly higher levels of the collagen receptor GPVI, whereas the receptor PEAR1 showed lower levels in COVID-19. These findings indicate the presence of a hyperactivated phenotype of platelets during SARS-CoV-2 infection, which might contribute to the hypercoagulopathy observed in COVID-19 and might influence disease progression. The adhesion protein P-Selectin translocates to the plasma membrane upon activation and regulates platelet–leukocyte interactions resulting in activation of neutrophil integrins and inducing NETs formation^33,34^. Moreover, platelet–leukocyte interaction may trigger the tissue factor expression as recently described in severe COVID-19^35^. P-Selectin expression together with the upregulation of the integrins GPIIb (CD41) and GPIIIa (CD61), and the subunits of the von Willebrand receptor GPIbα and GPIX, known to regulate platelet–leukocyte interactions, may contribute to the COVID-19 inflammatory response^33,36^. Consistent with our data, Manne et al.^11^ recently reported a higher surface expression of P-Selectin and higher levels of circulating platelet–leukocyte aggregates in COVID-19 patents. Moreover, the study showed a faster platelet aggregation and increased spreading on fibrinogen and collagen in COVID-19 patients compared to controls. The higher surface expression of integrins and adhesion protein detected in our study may provide a first mechanistic explanation to these findings.

To further investigate platelet reactivity in COVID-19, we induced platelet activation with TRAP, which activates platelets by thrombin signaling. After activation, we detected significantly higher levels of platelet activation markers P-Selectin and GPIIb/GPIIIa complex but not LAMP-3 in COVID-19 patients compared to healthy controls. Interestingly, we observed a decreased activation capacity in platelets of COVID-19 patients compared to controls, suggesting that the chronic platelet activation during SARS-CoV-2 correlates with an altered reactivity upon stimuli, which is possibly due to an higher activation level at rest in COVID-19 (Fig. 4)^8,33^. Of note, CD40 ligand (CD154) expression did not provide informative data: signal increased after TRAP stimulation but we did not detected any differences among groups (Supplemental Fig. II).

Subgroups investigations using FlowSOM analysis detected some differences in platelet activation patterns between healthy donors and COVID-19 patients (Supplemental Fig. III). However, as shown in Fig. 1, we did not find any defined and distinct subgroups, highlighting the lower heterogeneity of platelets compared to other cells in peripheral blood. Nonetheless, the FlowSOM trees shown in Supplemental Fig. III report a different activation pattern in COVID-19 patients compared to controls involving different platelet subgroups. Further studies are needed, to dissect the role of platelet heterogeneity in COVID-19 platelet activation.

Although the pathophysiological mechanisms behind the high incidence of thromboembolic events in hospitalized COVID-19 patients remain unclear, our data describe with high resolution the presence of activated platelets, which may provide one explanation for COVID-19 coagulopathy and suggests platelet inhibition as a possible therapeutic option in COVID-19 patients. Our data are consistent with previous studies reporting an immunothrombotic dysregulation as a typical marker of SARS-CoV-2 infection^11,13^. However, the key drivers behind platelet activation in COVID-19 remain to be determined. SARS-CoV-2 tropism for thrombocytes has not been proven yet and platelet activation may be induced by infected endothelium as well as by the cytokine storm occurring during SARS-CoV-2 infection^37^. Clinical trials investigating empirically different anticoagulation schemes and antiplatelet therapies are ongoing worldwide, and may provide more insights concerning the clinical relevance of antithrombotic regimes for COVID-19 patients^38^.

A strength of our analysis is the simultaneous measurement in a healthy control group, minimizing the risk that the observed higher platelet activation in COVID-19 was due to procedural biases. In addition, we restricted our measurements to stable COVID-19 patients not requiring supported respiration or extracorporeal perfusion, which may induce non-disease associated platelet activation. A further strength of our study consists in the high-resolution achieved by our measurements using mass cytometry, avoiding the spectral limitation of flow cytometry and allowing the measurements of 21 markers at single-cell level with virtually no overlapping.

Limitations of this study consist in the limited number of patients and in its ex vivo observational nature: our research was limited to the phenotypical observation of platelet surface receptor expressions and we did not assess the pathophysiological mechanisms triggering platelet activation. In fact, other pathways including the cytokine storm and the pro-inflammatory state during SARS-CoV-2 infection may play a relevant role in COVID-19 coagulopathy. Moreover, we did not include patients with non-COVID-19 inflammation and/or other types of viremia (e.g. influenza or other respiratory viruses) as an additional control group. Thus, we cannot quantify the severity of platelet activation in COVID-19 comparing it with other pathological settings. Nevertheless, here we provide the first mass cytometric analysis of platelets in COVID-19 and our results provide the basis for further research regarding pathways of platelet activation in COVID-19 patients as well as for further investigations of platelet biology in other pathological settings.

In conclusion, mass cytometry of COVID-19 patients revealed higher expression levels of platelet activation markers and adhesion proteins compared to healthy controls. These findings provide new insights into COVID-19 coagulopathy and support research projects investigating antithrombotic and antiplatelet treatment regimes in COVID-19.

## Supplementary information

Supplemental file
