# Supplementary material for: SARS-CoV-2 infection is associated with a pro-thrombotic platelet phenotype
Source: Cell Death Dis. 2021 Jan 5;12(1):50. doi: 10.1038/s41419-020-03333-9 (PMC7790351; doi:10.1038/s41419-020-03333-9)
Supplement: Supplementary file 1 — Supplemental file [file 41419_2020_3333_MOESM1_ESM.docx]

**SUPPLEMENTAL MATERIAL**

**SARS-CoV-2 infection is associated with a pro-thrombotic platelet phenotype**

Dario Bongiovanni M.D.*^1,2,3^, Melissa Klug M.Sc *^1,2,4^, Olga Lazareva M.Sc^4^, Simon Weidlich M.D.^5^, Marina Biasi B.Sc ^1^, Simona Ursu Ph.D ^6^, Sarah Warth Ph.D^6^, Christian Buske M.D.^6,7^, Marina Lukas M.D.^5^, Christoph D. Spinner M.D.^5^, Moritz von Scheidt M.D.^2,8^, Gianluigi Condorelli Ph.D^3^, Jan Baumbach Ph.D^4^, Karl-Ludwig Laugwitz M.D.^1,2^, Markus List Ph.D.^4,†^ and Isabell Bernlochner M.D.^1,2,†^

* These authors contributed equally
^†^These authors jointly supervised this work

^1^ Technical University of Munich, School of Medicine, University hospital rechts der Isar, Department of Internal Medicine I, Munich, Germany

^2^ German Center for Cardiovascular Research (DZHK), Partner Site Munich Heart Alliance, Germany;

^3^ Department of Cardiovascular Medicine, Humanitas Clinical and Research Center IRCCS and Humanitas University, Rozzano, Milan, Italy

^4^ Chair of Experimental Bioinformatics, TUM School of Life Sciences Weihenstephan, Technical University of Munich, Munich, Germany;

^5^ Technical University of Munich, School of Medicine, University hospital rechts der Isar, Department of Internal Medicine II, Munich, Germany

^6^ Core Facility Cytometry, Ulm University Medical Faculty, Germany

^7^ CCC Ulm, Institute of Experimental Cancer Research, University Hospital Ulm, Germany

^8^ Deutsches Herzzentrum München, Cardiology, Technische Universität München, Munich, Germany;

Running title: platelet activation during SARS-CoV-2 infection

1. **Supplemental Results**

**Flowsom and clustering analysis**

FlowSOM allows to investigate subsets of platelets with similar phenotype that are not detectable at the UMAPs analysis (Figure 1). As platelets are less heterogenous than other cells from peripheral blood and specific markers for subgroups are lacking, clustering analysis is challenging and did not provide strong results. Supplemental Figure III shows the different expression patterns involved in the TRAP-stimulation process in the control group compared to COVID-19 patients. Non-stimulated platelets of healthy donors express activation markers P-Selectin and LAMP-3 mostly in clusters 1 and 2, whereas COVID-19 patients show the highest expression of these markers in cluster 4 (Supplemental Figure IIIA). Interestingly, cluster 1 is characterized by a significantly higher median expression of CD62P and CD41 as well as a significantly lower median expression of CD61 and CD107a compared to COVID-19 patients. Cluster 4, containing most of the activated cells in patients, is characterized by a significantly higher median expression of GPIIb/IIIa complex, CD47 and CD9 (*P* values and medians are shown in Supplemental Table III and IV).

After TRAP-stimulation the clusters in which the platelets are most activated also differ between control and patients. COVID-infected platelets show most activation, mirrored in the higher P-Selectin and LAMP-3 expression, in cluster 2, whereas the control group shows most activated platelets in clusters 4 and 5 (Supplemental Figure IIIB).

1. **Supplemental Figures**

**Supplemental Figure I:** Gating protocol


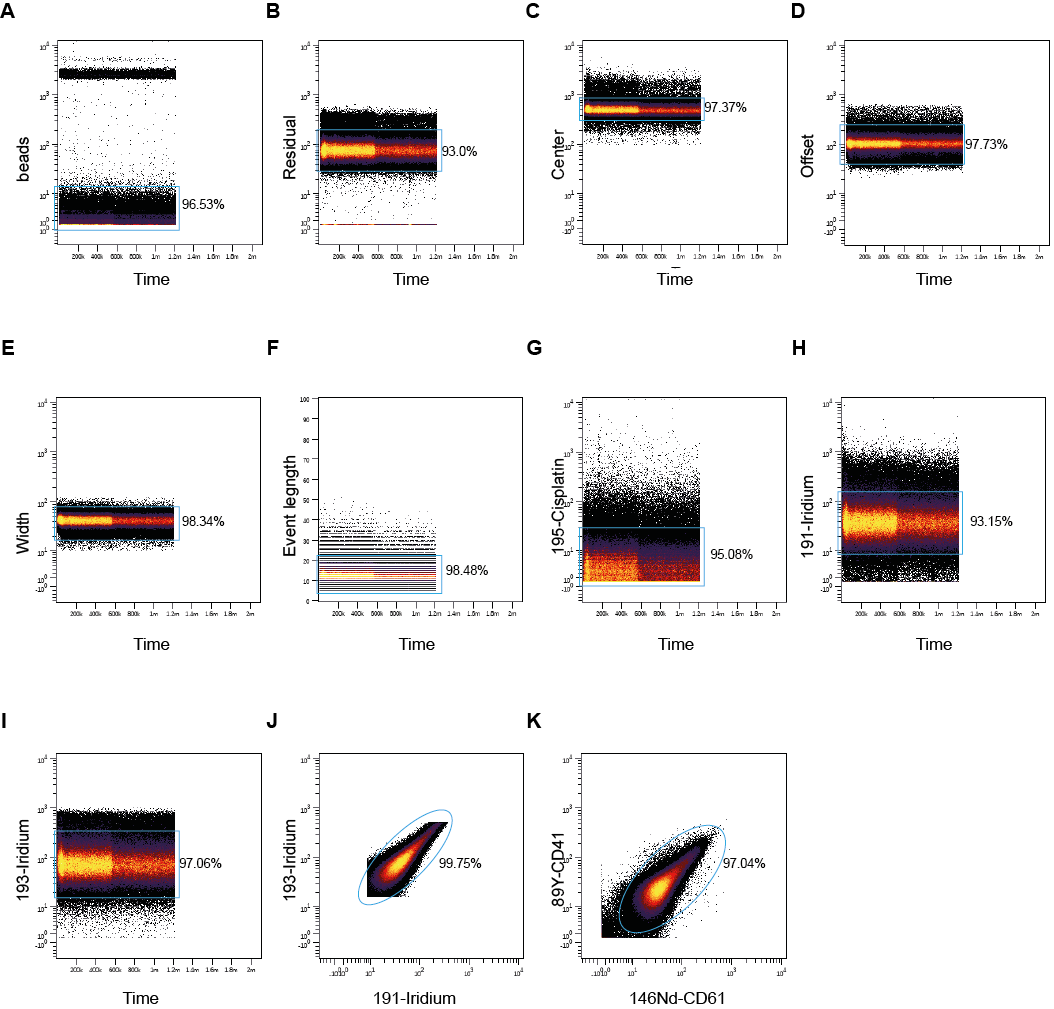
**Supplemental Figure I Legend: Gating protocol.** A) Exclusion of beads, B-E) clean-up according to the Gaussian discrimination, F) selecting event length for low range intensity, G) live-dead cell discrimination by only excluding cisplatin stained cells, H-J) RNA containing cells stained with 191/193-Iridium, K) gating only CD41 and CD61 positive cells to exclude debris and contamination.

**Supplemental Figure II:** Marker expression in non-stimulated and TRAP-stimulated platelets


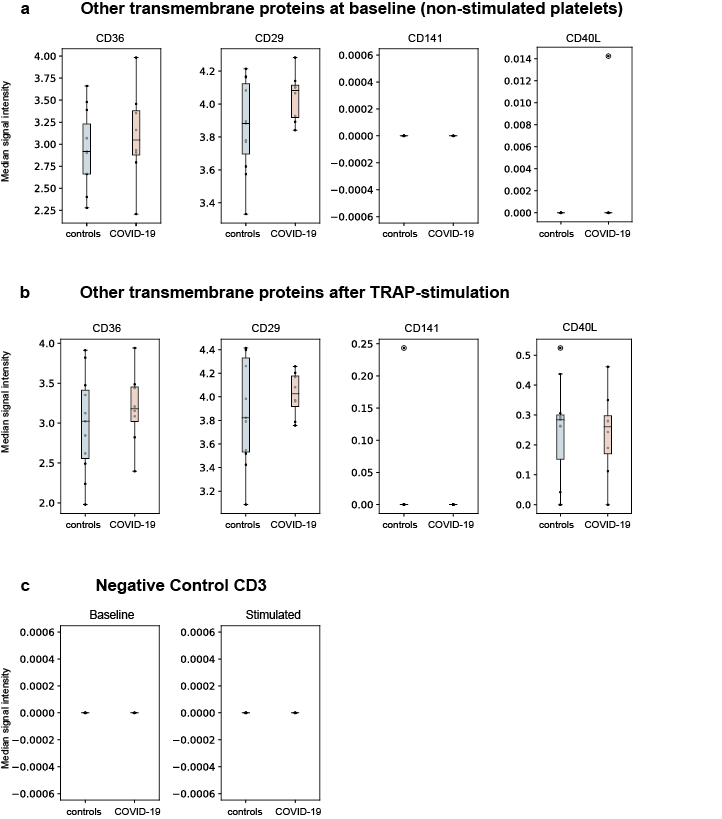


**Supplemental Figure II Legend: Marker expression in non-stimulated and TRAP-stimulated platelets.** Median signal intensity of transmembrane proteins measured in **(a)** non-stimulated platelets and **(b)** TRAP-stimulated platelets. **(c)** Negative control marker CD3 measured at baseline and after stimulation state. The horizontal line within the box plot represents the median, the top and bottom of each box indicate the interquartile range (Q1-Q3), whisker bars indicate the largest observation that is less than or equal to the upper inner fence (UIF =Q3+1.5*IQR) or the smallest observation that is greater than or equal to the lower inner fence (LIF=Q1-1.5*IQR). The circles indicate outliers, if present; *: p<0.01.

**Supplemental Figure III:** FlowSOM analysis in non-stimulated and TRAP-stimulated platelets


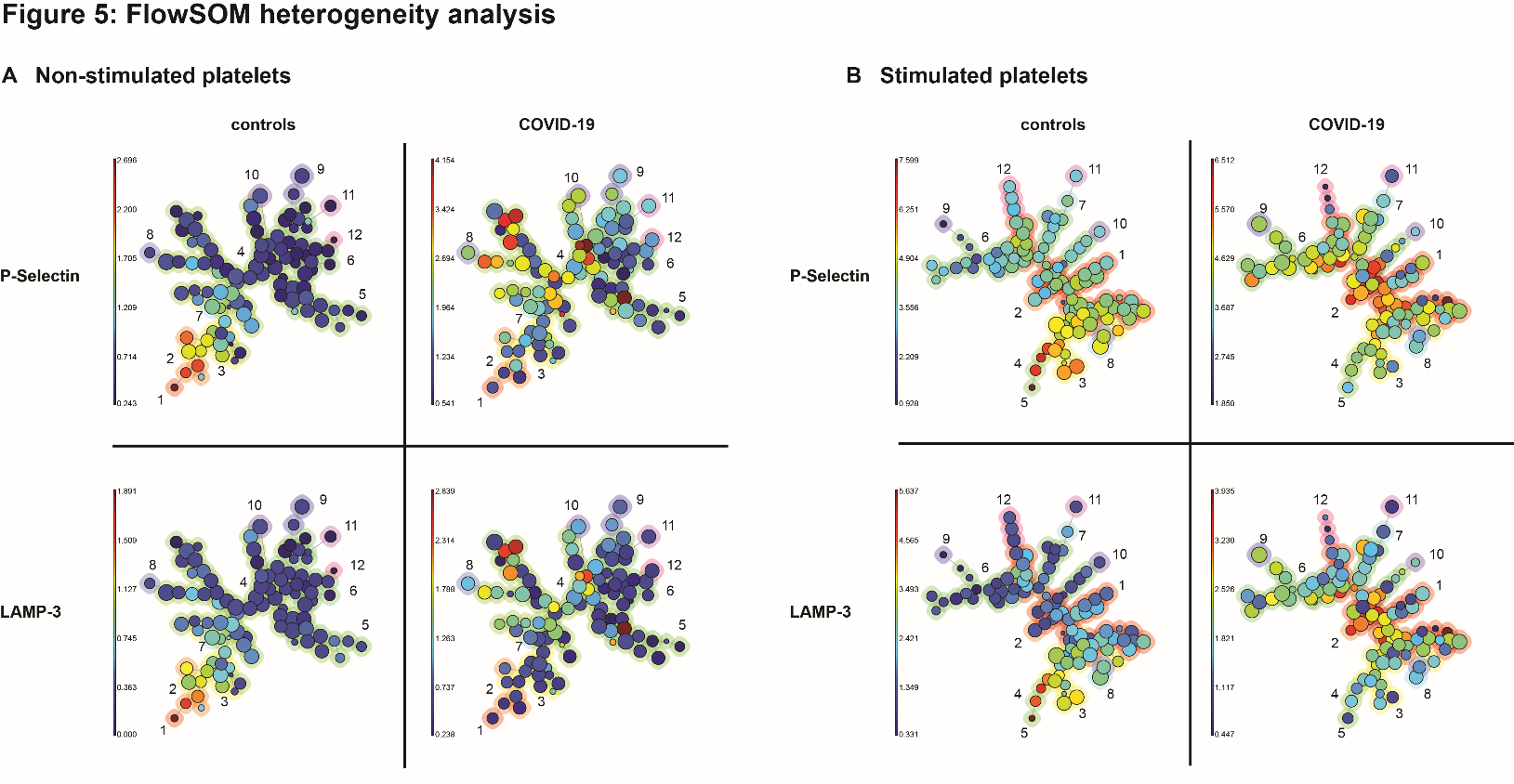


**Supplemental Figure III legend: Clustering analysis of platelets in healthy controls and COVID-19 patients.** Platelets from platelet-rich plasma were stained with the described CYTOF panel (see methods). Single live CD41/CD61 cells for each file were exported, concatenated and then analyzed using FlowSOM. Cells were clustered into nodes, which were divided over 12 metaclusters. This automatic meta-clustering of the FlowSOM nodes is indicated by the background color of the nodes (red, orange, yellow, lime, green, mint, cyan, azure, violet, purple, pink and magenta) and the numbers from 1-12. Separate FlowSOM trees show the P-Selectin (first row) and LAMP-3 (second row) expression across the clusters for healthy controls and COVID-19 patients in (A) non stimulated platelets and (B) after stimulation with 10 µM TRAP.

1. **Supplemental Tables**

**Supplemental Table I** Baseline demographic and clinical characteristics of the trial population

| Characteristic | Patients  (n=8) | Healthy donors  (n=11) | p value |
| --- | --- | --- | --- |
| Age (years), mean (+/-SD) | 51.4 (11.7) | 44.7 (13.0) | 0.27 |
| 18-39 years, no. (%) | 1 (12.5) | 4 (36) |  |
| 40-60 years, no. (%) | 5 (62.5) | 6 (55) |  |
| 60-70 years, no. (%) | 2 (25) | 1 (9) |  |
| Male sex, no. (%) | 5 (62.5) | 5 (45) | 0.49 |
| SARS-CoV-2 RT-PCR upper respiratory tract, no. (%) | 8 (100) |  |  |
| Oxygen saturation at admission, % mean (+/-SD) | 93.5 ± 2.9 |  |  |
| Length of hospital stay (days), mean (+/-SD) | 9.5 (6.3) |  |  |
| CT scan: typical lesions* no. (%) | 7 (88) |  |  |
| Thrombocytes (G/l), mean (+/-SD) | 225.5 (57.7) |  |  |
| Hemoglobin (g/dl), mean (+/-SD) | 13.1 (1.7) |  |  |
| Leukocytes (G/l), mean (+/-SD) | 4.9 (1.6) |  |  |
| CRP (mg/dl), mean (+/-SD) | 3.9 (7.1) |  |  |
| D-Dimer (µg/l), mean (+/-SD) | 728 (2294.2) |  |  |
| IL6 (pg/ml), mean (+/-SD) | 18.9 (21.2) |  |  |
| GFR (ml/min), mean (+/-SD) | 95.5 (17.6) |  |  |

Notes: In case of non-detectable level, the lowest detectable level of each parameter has been used for statistics: CRP < 0.1 is equal to 0.1, Il-6 <1.5 is equal to 1.5, D-Dimer <200 is equal to 200. SD: standard deviation. N=8 SARS-CoV-2 positive patients. *bilateral ground-glass opacities

**Supplemental Table II:**  CyTOF Antibodies and Reagents

| Antibody | Source | Identifier |
| --- | --- | --- |
| CD107a/LAMP1 (H4A3)-151Eu | Fluidigm Sciences | Cat#: 3151002B |
| CD141 (M80)166Er | Fluidigm Sciences | Cat#: 3166017B |
| CD154/CD40L (24-31)-168Er | Fluidigm Sciences | Cat#: 3168006B |
| CD29 (TS2/16)156Gd | Fluidigm Sciences | Cat#: 3156007B |
| CD3 (UCHT1)-170Er | Fluidigm Sciences | Cat#: 3170001B |
| CD31/PECAM-1 (WM59)-145Nd | Fluidigm Sciences | Cat#: 3145004B |
| CD36 (5-271)152Sm | Fluidigm Sciences | Cat#: 3152007B |
| CD40 (5C3)-142Nd | Fluidigm Sciences | Cat#: 3142010B |
| CD41 (HIP8)-89Y | Fluidigm Sciences | Cat#: 3089004B |
| CD42a (GR-P)- 141Pr | Thermofisher | Cat#: MA1-91023 |
| CD42b (HIP1)-144Nd | Fluidigm Sciences | Cat#: 3144020B |
| CD47 (CC2C6)209B | Fluidigm Sciences | Cat#: 3209004B |
| CD61 (VI-PL2)-146Nd | Fluidigm Sciences | Cat#: 3146011B |
| CD62P (KO-2-7)-161Dy | Thermofisher | Cat#: MA1-81809 |
| CD63 (H5C6)-150Nd | Fluidigm Sciences | Cat#: 3150021B |
| CD69 (FN50)162Dy | Fluidigm Sciences | Cat#: 3162001B |
| CD9 (SN4 C33A2)-171Yb | Fluidigm Sciences | Cat#: 3171009B |
| GPVI (HY101)-175Lu | Thermofisher | Cat#: 14-9813-81 |
| GPIIb/GPIIIa complex (CRC64)-155Gd | Biozol | Cat#: MBS465049 |
| PAR1 (ATAP2) -147Sm | Thermofisher | Cat#: 35-2200 |
| PEAR (492621)-174Yb | Novusbio | Cat#: MAB4527 |
| Maxpar® X8 Multimetal Labeling Kit | Fluidigm | Cat#: 201300 |
| Cell-ID™ Intercalator-Ir | Fluidigm | Cat#: 201192A |
| Cell-ID™ Cisplatin | Fluidigm | Cat#: 201064 |
| Maxpar® Cell Staining Buffer | Fluidigm | Cat#: 201068 |
| Maxpar® Fix and PermBuffer | Fluidigm | Cat#: 201067 |
| AB Stabilizer, PBS base | Boca Scientific | Cat#: 131050 |

**Supplemental Table III:**  *P* values compared using linear mixed effect model for the described clusters in non-stimulated platelets

| Marker | Cluster | *P* value | *P* value corrected | Median Control | Median COVID-19 |
| --- | --- | --- | --- | --- | --- |
| **CD61** | **1** | **<1x10^-250^** | **<1x10^-250^** | **4.789x10^14^** | **5.1643x10^14^** |
| **CD62P** | **1** | **<1x10^-250^** | **<1x10^-250^** | **3.2686x10^16^** | **4.573x10^14^** |
| **CD29** | **1** | **9.0205x10^-215^** | **1.9271x10^-213^** | **6.2913x10^15^** | **6.144x10^15^** |
| **CD41** | **1** | **4.1299x10^-10^** | **3.7328x10^-10^** | **4.407x10^15^** | **4.5827x10^14^** |
| **CD107a** | **1** | **3.7819x10^-7^** | **3.2917x10^-7^** | **3.3153x10^15^** | **7.4297x10^15^** |
| CD141 | 1 | 1.2438x10^9^ | 5.7312x10^8^ | 2.5689x10^14^ | 1.7287x10^16^ |
| CD45 | 1 | 1.3897x10^10^ | 5.9379x10^8^ | 2.5438x10^16^ | 2.1306x10^16^ |
| PEAR | 1 | 1.1527x10^11^ | 4.3691x10^10^ | 3.5706x10^16^ | 2.8945x10^16^ |
| CD42b | 1 | 4.9259x10^16^ | 5.8463x10^12^ | 5.6423x10^15^ | 5.8607x10^15^ |
| CD3 | 1 | 8.7116x10^15^ | 2.027x10^15^ | 2.8502x10^16^ | 1.1436x10^16^ |
| CD63 | 1 | 2.7188x10^16^ | 4.9915x10^15^ | 2.1707x10^16^ | 2.6622x10^15^ |
| CD47 | 1 | 5.5995x10^15^ | 6.5467x10^15^ | 4.8086x10^14^ | 4.9879x10^15^ |
| PAR1 | 1 | 6.5115x10^15^ | 7.3461x10^15^ | 3.6313x10^14^ | 3.4258x10^13^ |
| CD69 | 1 | 7.8502x10^15^ | 8.3975x10^15^ | 4.0856x10^15^ | 4.1516x10^15^ |
| CD42a | 1 | 7.9185x10^15^ | 8.3975x10^15^ | 5.8604x10^15^ | 5.855x10^15^ |
| CD40 | 1 | 8.2703x10^15^ | 8.7153x10^15^ | 1.2393x10^16^ | 1.2974x10^15^ |
| CD154 | 1 | 3.5171x10^16^ | 8.8873x10^15^ | 2.9581x10^15^ | 2.4996x10^15^ |
| GPIIb/GPIIIa complex | 1 | 7.3096x10^15^ | 1.1847x10^16^ | 7.0382x10^15^ | 7.235x10^15^ |
| GPVI | 1 | 6.1383x10^10^ | 2.2192x10^16^ | 5.6377x10^14^ | 5.3819x10^14^ |
| CD36 | 1 | 2.5488x10^16^ | 3.291x10^16^ | 5.4386x10^15^ | 5.4801x10^14^ |
| CD31 | 1 | 3.9753x10^15^ | 4.7663x10^16^ | 4.7019x10^15^ | 4.7062x10^15^ |
| CD9 | 1 | 3.3561x10^16^ | 6.0204x10^16^ | 6.6857x10^16^ | 6.8132x10^15^ |
| **PEAR** | **2** | **<1x10^-250^** | **<1x10^-250^** | **2.7487x10^16^** | **2.2745x10^16^** |
| **CD31** | **2** | **2.6082x10^-5^** | **0.0002** | **3.6938x10^16^** | **3.8553x10^16^** |
| CD45 | 2 | 23646.6 | 1.4624x10^6^ | 1.6076x10^16^ | 1.4287x10^14^ |
| CD42b | 2 | 66550 | 3.9099x10^6^ | 4.7507x10^15^ | 5.0019x10^15^ |
| CD107a | 2 | 6.8117x10^15^ | 2.0522x10^14^ | 2.2649x10^16^ | 3.7987x10^14^ |
| GPIIb/GPIIIa complex | 2 | 2.5182x10^16^ | 4.7343x10^14^ | 6.0753x10^15^ | 6.4499x10^15^ |
| CD141 | 2 | 6.7644x10^15^ | 1.1039x10^15^ | 0 | 0 |
| CD47 | 2 | 5.2213x10^15^ | 1.2916x10^15^ | 3.9796x10^14^ | 4.2452x10^14^ |
| CD69 | 2 | 6.6305x10^16^ | 1.5582x10^15^ | 3.1443x10^14^ | 3.4x10^15^ |
| CD63 | 2 | 7.466x10^15^ | 2.2208x10^15^ | 1.3572x10^14^ | 1.6128x10^15^ |
| PAR1 | 2 | 1.5633x10^16^ | 3.1945x10^15^ | 2.844x10^14^ | 2.6462x10^16^ |
| CD62P | 2 | 1.862x10^16^ | 3.6227x10^15^ | 2.3127x10^14^ | 3.2974x10^16^ |
| CD40 | 2 | 2.5809x10^16^ | 4.7757x10^15^ | 6.1466x10^14^ | 6.7899x10^15^ |
| CD61 | 2 | 1.9377x10^16^ | 6.5051x10^15^ | 3.8149x10^14^ | 4.3428x10^15^ |
| CD29 | 2 | 8.7437x10^14^ | 9.0919x10^15^ | 5.3786x10^15^ | 5.3715x10^16^ |
| CD154 | 2 | 9.1399x10^15^ | 9.3386x10^15^ | 2.3948x10^13^ | 1.646x10^15^ |
| CD3 | 2 | 1.4562x10^16^ | 2.093x10^16^ | 1.1879x10^14^ | 4.6158x10^15^ |
| CD41 | 2 | 9.4588x10^15^ | 2.117x10^16^ | 3.3999x10^14^ | 3.8161x10^16^ |
| GPVI | 2 | 1.0928x10^16^ | 2.4226x10^16^ | 4.9204x10^15^ | 4.7187x10^14^ |
| CD36 | 2 | 1.9802x10^16^ | 2.6899x10^16^ | 4.399x10^16^ | 4.6892x10^14^ |
| CD42a | 2 | 1.2363x10^15^ | 2.7152x10^16^ | 4.983x10^15^ | 5.0986x10^15^ |
| CD9 | 2 | 1.6483x10^15^ | 3.2551x10^16^ | 5.8293x10^15^ | 6.036x10^14^ |
| **GPIIb/GPIIIa complex** | **4** | **<1x10^-250^** | **<1x10^-250^** | **4.5805x10^14^** | **5.0348x10^15^** |
| **CD47** | **4** | **<1x10^-250^** | **<1x10^-250^** | **2.366x10^14^** | **2.707x10^16^** |
| **CD9** | **4** | **3.2894x10^-181^** | **6.4418x10^-179^** | **4.3633x10^14^** | **4.668x10^15^** |
| CD61 | 4 | 6.5894x10^5^ | 3.6869x10^7^ | 2.3594x10^14^ | 2.8277x10^16^ |
| PEAR | 4 | 2.0109x10^7^ | 9.8449x10^7^ | 1.5474x10^16^ | 1.2398x10^14^ |
| CD45 | 4 | 2.5591x10^7^ | 1.3986x10^8^ | 5.6262x10^14^ | 5.0433x10^15^ |
| CD42b | 4 | 9.4779x10^8^ | 4.2025x10^9^ | 3.2911x10^14^ | 3.6601x10^15^ |
| CD29 | 4 | 7.3992x10^15^ | 8.0875x10^14^ | 3.9467x10^14^ | 3.9979x10^16^ |
| CD62P | 4 | 6.3814x10^15^ | 1.5302x10^15^ | 5.9289x10^14^ | 1.249x10^15^ |
| CD31 | 4 | 1.7487x10^16^ | 2.4461x10^15^ | 2.2297x10^16^ | 2.4554x10^15^ |
| CD40 | 4 | 1.3482x10^16^ | 2.8803x10^15^ | 0 | 0 |
| GPVI | 4 | 2.3007x10^16^ | 4.3957x10^15^ | 3.4869x10^14^ | 3.2768x10^15^ |
| PAR1 | 4 | 3.8031x10^16^ | 4.6068x10^15^ | 1.4133x10^16^ | 1.2348x10^15^ |
| CD69 | 4 | 5.7294x10^15^ | 6.6654x10^15^ | 1.953x10^16^ | 2.0041x10^16^ |
| CD41 | 4 | 3.2817x10^15^ | 8.4035x10^15^ | 1.8969x10^16^ | 2.3588x10^14^ |
| CD42a | 4 | 3.041x10^16^ | 9.9256x10^15^ | 3.5568x10^16^ | 3.6519x10^16^ |
| CD36 | 4 | 1.0721x10^16^ | 1.6908x10^16^ | 2.9472x10^14^ | 3.2089x10^16^ |
| CD63 | 4 | 1.0902x10^16^ | 1.6967x10^16^ | 3.6263x10^14^ | 4.7517x10^15^ |
| CD107a | 4 | 1.8926x10^15^ | 3.6456x10^16^ | 0 | 0 |

**Supplemental Table IV:**  *P* values compared using linear mixed effect model for the described clusters in stimulated platelets

| Marker | Cluster | *P* value | *P* value corrected | Median Control | Median COVID-19 |
| --- | --- | --- | --- | --- | --- |
| CD36 | 2 | 8.8872x10^15^ | 9.1779x10^13^ | 3.2686x10^14^ | 3.3664x10^16^ |
| CD29 | 2 | 1.0197x10^16^ | 2.5609x10^14^ | 4.1338x10^14^ | 3.8753x10^14^ |
| GPVI | 2 | 1.0088x10^16^ | 2.5609x10^14^ | 3.4052x10^16^ | 3.1317x10^14^ |
| CD47 | 2 | 4.8015x10^15^ | 6.0636x10^14^ | 2.6911x10^16^ | 2.9689x10^16^ |
| CD62P | 2 | 6.5394x10^15^ | 7.4496x10^14^ | 4.1018x10^15^ | 4.3811x10^16^ |
| CD69 | 2 | 2.584x10^14^ | 3.8848x10^15^ | 1.9053x10^16^ | 2.0767x10^16^ |
| CD42b | 2 | 2.6123x10^16^ | 3.8956x10^15^ | 3.3737x10^16^ | 3.4955x10^14^ |
| CD107a | 2 | 4.2718x10^16^ | 5.601x10^15^ | 2.6415x10^16^ | 2.5124x10^16^ |
| CD3 | 2 | 4.8504x10^16^ | 6.0906x10^15^ | 0 | 0 |
| PAR1 | 2 | 5.1036x10^13^ | 6.3365x10^15^ | 4.5664x10^15^ | 4.2549x10^16^ |
| CD31 | 2 | 5.7245x10^15^ | 6.7674x10^15^ | 2.5203x10^15^ | 2.4991x10^16^ |
| CD141 | 2 | 5.8479x10^15^ | 6.7674x10^15^ | 0 | 0 |
| CD9 | 2 | 6.7705x10^15^ | 7.6732x10^15^ | 4.5782x10^16^ | 4.6065x10^14^ |
| CD42a | 2 | 6.9407x10^13^ | 7.826x10^15^ | 3.5087x10^15^ | 3.5515x10^16^ |
| CD40 | 2 | 7.2491x10^15^ | 8.0103x10^15^ | 0 | 0 |
| GPIIb/GPIIIa complex | 2 | 7.6033x10^15^ | 8.2775x10^15^ | 5.0073x10^15^ | 5.0389x10^14^ |
| CD154 | 2 | 8.6336x10^14^ | 8.9686x10^15^ | 1.9048x10^15^ | 2.0018x10^16^ |
| CD41 | 2 | 9.7838x10^15^ | 9.9185x10^15^ | 2.3461x10^16^ | 2.4627x10^14^ |
| PEAR | 2 | 7.1384x10^15^ | 1.4473x10^16^ | 1.5801x10^15^ | 1.3833x10^14^ |
| CD61 | 2 | 1.3435x10^16^ | 3.2991x10^16^ | 2.6533x10^16^ | 3.1428x10^14^ |
| CD63 | 2 | 2.1906x10^16^ | 3.4899x10^16^ | 1.8883x10^13^ | 1.7499x10^16^ |
| **CD41** | **4** | **8.4064x10^-7^** | **0.0001** | **4.4365x10^15^** | **4.5144x10^14^** |
| CD47 | 4 | 78.551 | 7890.9 | 4.8513x10^15^ | 4.8159x10^15^ |
| PEAR | 4 | 1.3555x10^6^ | 1.1095x10^7^ | 3.5899x10^14^ | 2.6513x10^16^ |
| CD141 | 4 | 2.3663x10^8^ | 1.5847x10^8^ | 4.5298x10^14^ | 1.7541x10^16^ |
| PAR1 | 4 | 1.2052x10^9^ | 7.1986x10^8^ | 3.4496x10^15^ | 3.0544x10^16^ |
| CD29 | 4 | 5.0706x10^8^ | 2.8015x10^9^ | 6.1473x10^16^ | 5.8864x10^16^ |
| CD42a | 4 | 3.0415x10^15^ | 9.2078x10^14^ | 5.571x10^14^ | 5.3545x10^15^ |
| CD3 | 4 | 4.0944x10^10^ | 1.7401x10^15^ | 2.8731x10^14^ | 1.8212x10^14^ |
| CD69 | 4 | 2.6611x10^16^ | 3.8956x10^15^ | 4.1835x10^15^ | 4.0367x10^15^ |
| CD31 | 4 | 1.733x10^16^ | 4.1164x10^15^ | 4.4233x10^15^ | 4.2745x10^15^ |
| GPIIb/GPIIIa complex | 4 | 4.7583x10^14^ | 6.0435x10^15^ | 7.0584x10^13^ | 7.0418x10^15^ |
| CD107a | 4 | 5.2382x10^15^ | 6.3785x10^15^ | 1.6701x10^14^ | 1.9428x10^15^ |
| CD40 | 4 | 2.6649x10^15^ | 9.8157x10^15^ | 1.1691x10^14^ | 1.0274x10^15^ |
| CD9 | 4 | 9.8464x10^15^ | 9.9363x10^15^ | 6.6826x10^15^ | 6.6441x10^15^ |
| CD62P | 4 | 5.3384x10^15^ | 1.4747x10^16^ | 6.7002x10^13^ | 6.3378x10^16^ |
| CD154 | 4 | 8.7294x10^15^ | 1.7073x10^16^ | 1.062x10^14^ | 7.3675x10^15^ |
| CD63 | 4 | 5.4837x10^14^ | 1.9237x10^16^ | 4.6271x10^14^ | 3.8839x10^15^ |
| CD36 | 4 | 1.0764x10^16^ | 2.016x10^16^ | 5.1902x10^14^ | 5.1622x10^16^ |
| CD42b | 4 | 1.3156x10^15^ | 2.3684x10^16^ | 5.4886x10^15^ | 5.3726x10^14^ |
| CD61 | 4 | 1.5982x10^15^ | 2.738x10^16^ | 4.687x10^15^ | 4.9245x10^15^ |

**Supplemental Table V:** Antibodies

| Target antigen | Vendor or Source | Catalog # | Working concentration | Persistent ID / URL |
| --- | --- | --- | --- | --- |
| CD62P | Thermofisher | MA1-81809 | 0.5 mg/ml | https://www.thermofisher.com/antibody/product/P-Selectin-Antibody-clone-Psel-KO-2-7-Monoclonal/MA1-81809 |
| PEAR | Novusbio | MAB4527 | 0.5 mg/ml | https://www.novusbio.com/products/pear1-antibody-492621_mab4527 |
| PAR1 | Thermofisher | 35-2200 | 0.5 mg/ml | https://www.thermofisher.com/antibody/product/PAR1-Antibody-clone-ATAP2-Monoclonal/35-2200 |
| CD42a | Thermofisher | MA1-91023 | 0.5 mg/ml | https://www.thermofisher.com/antibody/product/CD42a-Antibody-clone-GR-P-Monoclonal/MA1-91023 |
| GPIIb/GPIIIa complex | Biozol | MBS465049 | 0.5 mg/ml | https://www.mybiosource.com/monoclonal-dog-human-rabbit-antibody/cd41-cd61/465049 |
| GPVI | Thermofisher | 14-9813-81 | 0.5 mg/ml | https://www.thermofisher.com/antibody/product/GP6-Antibody-clone-HY101-Monoclonal/14-9813-81 |
| CD107a/LAMP1 | Fluidigm Sciences | 3151002B | 0.5 mg/ml | https://www.fluidigm.com/reagents/proteomics/3151002b-antihuman-cd107a-lamp1--h4a3--151eu-^-10^0tests |
| CD141 | Fluidigm Sciences | 3166017B | 0.5 mg/ml | https://www.fluidigm.com/reagents/proteomics/3166017b-antihuman-cd141--m80--166er-^-10^0tests |
| CD154/CD40L | Fluidigm Sciences | 3168006B | 0.5 mg/ml | https://www.fluidigm.com/reagents/proteomics/3168006b-antihuman-cd154-cd40l--24-31--168er-^-10^0tests |
| CD29 | Fluidigm Sciences | 3156007B | 0.5 mg/ml | https://www.fluidigm.com/reagents/proteomics/3156007b-antihuman-cd29--ts2-16--156gd-^-10^0tests |
| CD3 | Fluidigm Sciences | 3170001B | 0.5 mg/ml | https://www.fluidigm.com/reagents/proteomics/3170001b-antihuman-cd3--ucht1--170er-^-10^0tests |
| CD31/PECAM | Fluidigm Sciences | 3145004B | 0.5 mg/ml | https://www.fluidigm.com/reagents/proteomics/3145004b-antihuman-cd31-pecam-1--wm59--145nd-^-10^0tests |
| CD36 | Fluidigm Sciences | 3152007B | 0.5 mg/ml | https://www.fluidigm.com/reagents/proteomics/3152007b-antihuman-cd36--5-271--152sm-^-10^0tests |
| CD40 | Fluidigm Sciences | 3142010B | 0.5 mg/ml | https://www.fluidigm.com/reagents/proteomics/3142010b-antihuman-cd40--5c3--142nd-^-10^0tests |
| CD41 | Fluidigm Sciences | 3089004B | 0.5 mg/ml | https://www.fluidigm.com/reagents/proteomics/3089004b-antihuman-cd41--hip8--89y-^-10^0tests |
| CD42b | Fluidigm Sciences | 3144020B | 0.5 mg/ml | https://www.fluidigm.com/reagents/proteomics/3144020b-antihuman-cd42b--hip1--144nd-^-10^0tests |
| CD47 | Fluidigm Sciences | 3209004B | 0.5 mg/ml | https://www.fluidigm.com/reagents/proteomics/3209004b-antihuman-cd47--cc2c6--209bi-^-10^0tests |
| CD61 | Fluidigm Sciences | 3146011B | 0.5 mg/ml | https://www.fluidigm.com/reagents/proteomics/3146011b-antihuman-cd61--vi-pl2--146nd-^-10^0tests |
| CD63 | Fluidigm Sciences | 3150021B | 0.5 mg/ml | https://www.fluidigm.com/reagents/proteomics/3150021b-antihuman-cd63--h5c6--150nd-^-10^0tests |
| CD69 | Fluidigm Sciences | 3162001B | 0.5 mg/ml | https://www.fluidigm.com/reagents/proteomics/3162001b-antihuman-cd69--fn50--162dy-^-10^0tests |
| CD9 | Fluidigm Sciences | 3171009B | 0.5 mg/ml | https://www.fluidigm.com/reagents/proteomics/3171009b-antihuman-cd9--sn4-c3-3a2--171yb-^-10^0tests |
